# Zoonotic Microsporidia in Wild Lagomorphs in Southern Spain

**DOI:** 10.3390/ani10122218

**Published:** 2020-11-26

**Authors:** Anabel Martínez-Padilla, Javier Caballero-Gómez, Ángela Magnet, Félix Gómez-Guillamón, Fernando Izquierdo, Leonor Camacho-Sillero, Saúl Jiménez-Ruiz, Carmen del Águila, Ignacio García-Bocanegra

**Affiliations:** 1 Departamento de Biología Molecular y Bioquímica, Universidad de Málaga (UMA), 29010 Málaga, Spain; anabelmp@uma.es; 2 Grupo de Investigación en Sanidad Animal y Zoonosis (GISAZ), Departamento de Sanidad Animal, Universidad de Córdoba (UCO), 14014 Córdoba, Spain; sauljimenezruiz@gmail.com (S.J.-R.); nacho.garcia@uco.es (I.G.-B.); 3 Unidad de Enfermedades Infecciosas, Grupo de Virología Clínica y Zoonosis, Instituto Maimónides de Investigación Biomédica de Córdoba (IMIBIC), Universidad de Córdoba (UCO), 14004 Córdoba, Spain; 4 Facultad de Farmacia, Universidad San Pablo-CEU, CEU Universities, Urbanización Montepríncipe, Boadilla del Monte, 28660 Madrid, Spain; angela.magnetdavila@ceu.es (Á.M.); ferizqui@ceu.es (F.I.); cagupue@ceu.es (C.d.Á.); 5 Programa de Vigilancia Epidemiológica de la Fauna Silvestre en Andalucía (PVE), Consejería de Agricultura, Ganadería, Pesca y Desarrollo Sostenible, Junta de Andalucía, 29006 Málaga, Spain; felixj.gomezguillamon@juntadeandalucia.es (F.G.-G.); leonorn.camacho@juntadeandalucia.es (L.C.-S.); 6 Grupo Sanidad y Biotecnología (SaBio), Instituto de Investigación en Recursos Cinegéticos (IREC-CSIC-JCCM), Universidad de Castilla-la Mancha (UCLM), 13005 Ciudad Real, Spain

**Keywords:** *Encephalitozoon intestinalis*, *E. hellem*, *E. cuniculi*, *Enterocytozoon bieneusi*, European wild rabbit, Iberian hare, zoonotic, foodborne

## Abstract

**Simple Summary:**

A cross-sectional study was carried out to assess the presence of zoonotic microsporidia in organ meats of European wild rabbits and Iberian hares consumed by humans in Spain. Between July 2015 and December 2018, kidney samples from 383 wild rabbits and kidney and brain tissues from 79 Iberian hares in southern Spain were tested by species-specific polymerase chain reactions (PCRs) for the detection of microsporidia DNA. We confirmed the presence of *Enterocytozoon bieneusi* in three wild rabbits and *Encephalitozoon intestinalis* in one wild rabbit and three Iberian hares. However, none of the 462 sampled wild lagomorphs showed *Encephalitozoon hellem* nor *Encephalitozoon cuniculi* infection. This is the first report of *E. intestinalis* infection in wild rabbits and Iberian hares. The presence of *E. bieneusi* and *E. intestinalis* in organ meats from wild lagomorphs can be of public health concern. Additional studies are required to determine the real prevalence of these parasites in European wild rabbit and Iberian hare.

**Abstract:**

Microsporidia are obligate intracellular protist-like fungal pathogens that infect a broad range of animal species, including humans. This study aimed to assess the presence of zoonotic microsporidia (*Enterocytozoon bieneusi*, *Encephalitozoon intestinalis*, *Encephalitozoon hellem*, and *Encephalitozoon cuniculi*) in organ meats of European wild rabbit (*Oryctolagus cuniculus*) and Iberian hare (*Lepus granatensis*) consumed by humans in Spain. Between July 2015 and December 2018, kidney samples from 383 wild rabbits and kidney and brain tissues from 79 Iberian hares in southern Spain were tested by species-specific PCR for the detection of microsporidia DNA. *Enterocytozoon bieneusi* infection was confirmed in three wild rabbits (0.8%; 95% CI: 0.0–1.7%) but not in hares (0.0%; 95% CI: 0.0–4.6%), whereas *E. intestinalis* DNA was found in one wild rabbit (0.3%; 95% CI: 0.0–0.8%) and three Iberian hares (3.8%; 95% CI: 0.0–8.0%). Neither *E. hellem* nor *E. cuniculi* infection were detected in the 462 (0.0%; 95% CI: 0.0–0.8%) lagomorphs analyzed. The absence of *E. hellem* and *E. cuniculi* infection suggests a low risk of zoonotic foodborne transmission from these wild lagomorph species in southern Spain. To the authors’ knowledge, this is the first report of *E. intestinalis* infection in wild rabbits and Iberian hares. The presence of *E. bieneusi* and *E. intestinalis* in organ meats from wild lagomorphs can be of public health concern. Additional studies are required to determine the real prevalence of these parasites in European wild rabbit and Iberian hare.

## 1. Introduction

The European wild rabbit (*Oryctolagus cuniculus*) and the Iberian hare (*Lepus granatensis*) are two endemic species in the Iberian Peninsula. Both lagomorph species play a key role in the ecology of Mediterranean ecosystems [1,2]. They are the staple prey for a large number of predators, including endangered species such as the Iberian lynx (*Lynx pardinus*) and the Spanish imperial eagle (*Aquila adalberti*) [3]. These lagomorphs are also among the main small game species in Spain. About 5.3 million wild rabbits and 890 thousand Iberian hares are harvested annually in this country and are generally consumed without sanitary inspection since they are intended for small-scale retail sale or personal consumption [4]. Public health concerns indicate the need for epidemiological studies on zoonotic diseases affecting wildlife species that are a source of food for humans [5]. In this respect, the role of wild lagomorphs as reservoirs of zoonotic parasites has been widely documented [6,7,8].

The phylum Microsporidia comprises more than 1500 obligate intracellular spore-forming parasites classified as fungi that can infect a wide range of vertebrate and invertebrate hosts through the fecal–oral route [9]. Among the 17 zoonotic microsporidia species, *Enterocytozoon bieneusi, Encephalitozoon intestinalis, E. hellem,* and *E. cuniculi* are the most common species reported in humans [10,11]. Microsporidiosis in humans is usually characterized by chronic diarrhea and wasting syndrome, although keratoconjunctivitis, hepatitis, myositis, kidney and urogenital infection, ascites, and/or cholangitis have also been reported, particularly in immunocompromised individuals [12].

Foodborne transmission has previously been evidenced as a zoonotic route of microsporidia infection [13]. In this regard, in European countries, zoonotic microsporidia species have been detected in the Eastern cottontail rabbit (*Sylvilagus floridanus*), the European brown hare (*Lepus europaeus*), and the European rabbit [14,15,16,17]. Even though microsporidia infections in these lagomorph species are usually asymptomatic, microsporidiosis has been reported in domestic rabbit and European brown hare, causing neurological and/or renal disorders and even death [17,18,19]. However, knowledge about the role of wild lagomorphs in the epidemiology of microsporidia remains scarce [20]. Hence, the present study aimed to assess the presence of *E. bieneusi, E. intestinalis, E. hellem,* and *E. cuniculi* in organ meats from European wild rabbit and Iberian hare, the most frequent wild lagomorph species consumed by humans in Spain.

## 2. Materials and Methods

**Figure 1:**
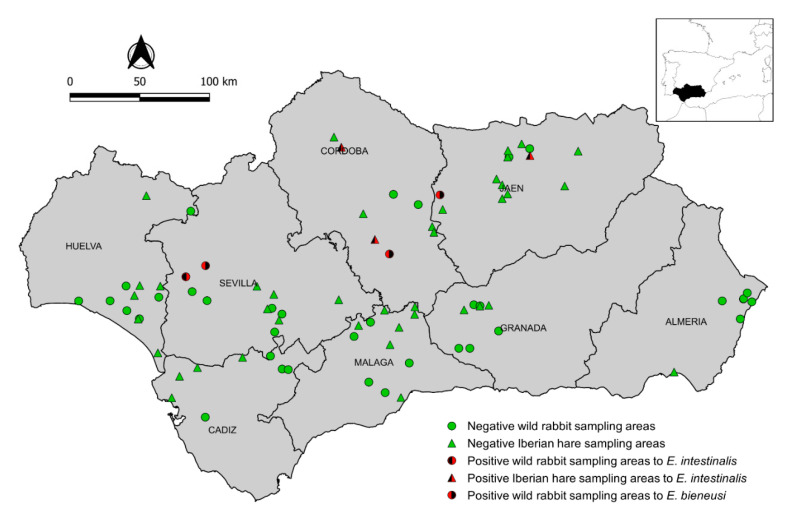
Map of Andalusia (southern Spain) showing the locations of European wild rabbits (dots) and Iberian hares (triangles) sampled. Green and red symbols indicate negative and positive animals, respectively, detected in these hunting areas.

A cross-sectional study was carried out on wild lagomorphs in Andalusia (southern Spain) (36° N–38°60′ N, 1°75′ W–7°25′ W, 87,268 km^2^) between July 2015 and December 2018. The sample size for wild rabbits was calculated assuming a prevalence of 50% (the highest sample size for studies with unknown prevalence) with a 95% confidence level (95% CI) and a desired precision of ±5% [21], which gave 384 wild rabbits to be sampled. Whenever possible, a minimum of 49 animals were sampled in each of the eight provinces of Andalusia in order to detect microsporidia infection with a 95% probability and an assumed minimum within-province prevalence of 6%. Kidney samples were collected from 383 wild rabbits in 40 hunting areas (Figure 1). In the same study region and period, kidney and brain samples were also obtained from 79 Iberian hares in 43 hunting or protected areas (Figure 1). Sampled animals were necropsied in the field. Samples were placed in sterile bottles (25 mL), preserved at 4 °C while transported to the laboratory, and stored at −20 °C until analysis. Data on species, age, gender, sampling site, province, and sampling year were gathered from each wild lagomorph sampled, whenever possible. Data analyses were carried out using the SPSS statistical software package version 25.0 (IBM Corporation, Somers, NY, USA). 

This study did not involve purposeful killing of animals. The collection of samples was performed by personnel of the Epidemiological Surveillance Program in Wildlife of the Government of Andalusia. Samples were collected from legally hunted animals during the hunting seasons or by passive surveillance in complete agreement with Andalusian, Spanish, and European regulations. No ethical approval by an Institutional Animal Care and Use Committee was deemed necessary.

Genomic DNA was extracted from the kidney and brain samples using the G-spin™ total DNA extraction kit (Intron Biotechnology, Seongnam-Si, Korea), following the manufacturer’s instructions. PCR amplifications were performed using the GenAmp kit (Perkin-Elmer Cetus, Norwalk, CT, USA) to detect small-subunit rRNA (SSU rRNA) coding regions of the microsporidia using species-specific primers previously described (Table 1) in a Gene Amp PCR system 9700 thermocycler (Perkin Elmer). The final concentration was 0.2 mM of each dNTP, 0.2 µM of each primer, buffer with MgCl_2_ (1.5 mM), and 1.25 U of Taq polymerase. Conditions for PCR reactions were as follows: denaturing at 94 °C for 30 s followed by 35 cycles of annealing at 45 °C for 30 s for *E. intestinalis* primers or 55 °C for 30 s for the remaining microsporidia species and extension at 72 °C for 90 s. PCR products were analyzed by electrophoresis in 2% agarose gel stained with ethidium bromide and examined under UV light.

## 3. Results

Microsporidia infection was detected in 7 of the 462 (1.5%; 95% CI: 0.4–2.6) wild lagomorphs analyzed. The distribution of prevalence according to species, province, year, age, and sex is shown in Table 2. Four of the 383 (1.0%; 95% CI: 0.0–2.1) wild rabbits and 3 of the 79 (3.8%; 95% CI: 0.0–8.0) Iberian hares tested positive for microsporidia infection. Positive animals were found in 7 of 83 (8.4%) sampling areas and 3 of 8 (37.5%) provinces (Figure 1). *E. bieneusi* infection was detected in wild rabbits (3/383; 0.8%; 95% CI: 0.0–1.7) but not in Iberian hares (0/79; 0.0%; 95% CI: 0.0–4.6). *E. intestinalis* was confirmed in one wild rabbit (0.3%; 95% CI: 0.0–0.8) and in two brain and one kidney samples from three Iberian hares (3.8%; 95% CI: 0.0–8.0). Co-infections with *E. bieneusi* and *E. intestinalis* were not observed. None of the 462 (0.0%; 95% CI: 0.0–0.8) wild lagomorphs showed positive results for *E. hellem* or *E. cuniculi* infection in the examined organs.

## 4. Discussion

This is the first large-scale study on zoonotic microsporidia species, including *E. bieneusi, E. intestinalis, E. hellem*, and *E. cuniculi*, in wild rabbits in the Iberian Peninsula and also the first report of microsporidia infection in Iberian hares worldwide. The presence of *E. bieneusi* infection in wild rabbits is consistent with that previously documented [26] and confirms the susceptibility of European wild rabbits to this pathogen. *E. bieneusi* infection has also been detected in farmed rabbits [27,28,29]. The low prevalence of this parasite detected in organ meats from wild rabbits and the absence of positivity in Iberian hares suggest that these mammal species could act as a spillover host rather than a true reservoir of this emerging pathogen. However, additional studies including fecal samples are required to determine the real prevalence of *E. bieneusi* in wild lagomorphs in the study area. In Spanish Mediterranean ecosystems, *E. bieneusi* infection has been detected in different sympatric species, including wild boar [30], red foxes (*Vulpes vulpes*), beech martens (*Martes foina*), and European badgers (*Meles meles*) [11,27], some of which can be predator species of wild lagomorphs. The high homology between *E. bieneusi* isolates obtained from humans, wild boar, and wild carnivores provides evidence of the zoonotic potential of wildlife [11,30]. In this regard, *E. bieneusi* infection has been reported in HIV/AIDS patients, organ transplant recipients, and immunocompetent individuals in Spain [31,32,33]. Further molecular studies are needed to assess the risk of zoonotic transmission of this pathogen from wild rabbits.

To the best of our knowledge, this is the first report of *E. intestinalis* infection in wild rabbits and Iberian hares, which increases the number of animal species susceptible to this microsporidian species. There is only one previous report of *E. intestinalis* infection in wild lagomorphs, particularly European brown hares [17]. *E. intestinalis* infection has been reported in humans in several European countries [34,35,36]. Even though this parasite has not been detected in humans in Spain so far, and the frequency of positive organ meat samples detected in the present study was low (0.3% and 3.8% in wild rabbits and Iberian hares, respectively), our results reveal *E. intestinalis* circulation in this country and, therefore, the risk of transmission of these food-borne zoonotic pathogen cannot be ruled out. Based on the number of animals infected with *E. bieneusi* or *E. intestinalis* found in the present study and hunting bag records for wild lagomorphs [4], around 53,000 wild rabbits and 33,800 Iberian hares infected with these microsporidia species may be consumed every year in Spain.

*E. hellem* or *E. cuniculi* DNA was not found in any of the 383 wild rabbits or 79 Iberian hares analyzed. These results are consistent with previous studies that also failed to detect these parasites in feces in wild and domestic rabbits [26,27,28,29] and in kidney and brain samples in domestic rabbits [35]. In contrast, DNA of *E. hellem* was confirmed in the kidneys of a free-living European brown hare with chronic interstitial nephritis [17]. In addition, *E. cuniculi* infections have been confirmed in kidney and brain tissues of free-living Eastern cottontail rabbits [15] as well as in farmed and pet European rabbits [37,38]. Serological evidence of *E. cuniculi* exposure has also been detected in wild rabbits in other European countries, with seroprevalence values ranging between 3.9% in France and 44.7% in Slovakia [16,39]. The absence of *E. cuniculi* infection in Iberian hares in our study is in accordance with previous observations in other hare species [37,40]. Likewise, seroprevalence values found in European brown hares ranged between 0.0% in Italy and 2.9% in the Czech Republic [14,41]. Further serosurvey studies are warranted to assess *E. hellem* and *E. cuniculi* circulation in the wild lagomorph populations in Spain.

Our study has some limitations that should be noted. Even though our objective was focused on assessing the risk of zoonotic foodborne transmission of the selected microsporidia species, fecal or duodenal tissue could be more appropriate samples for detecting infection of some of these pathogens. On the other hand, *E. intestinalis* DNA was detected in brain samples from Iberian hares, whose kidney tissues tested negative. Unfortunately, brain samples could not be collected from wild rabbits in the present study. While kidney tissue has been shown to be suitable for detection of microsporidia infection in wildlife species [42], including lagomorphs [17], previous observations have shown different sensitivities using brain and kidney samples [15,41,42]. Our results indicate that the prevalence of *E. intestinalis* obtained in wild rabbits in the present study, as well as those previously reported in other species, may be underestimated. To increase the sensitivity of microsporidia detection in organ meat samples, kidney and brain tissues should be tested in parallel.

## 5. Conclusions

The absence of positivity to *E. hellem* or *E. cuniculi* denotes a limited role of wild rabbits and Iberian hares in the zoonotic transmission of these microsporidia species in southern Spain. The detection of *E. bieneusi* in wild rabbits and *E. intestinalis* in both wild lagomorph species could be of public health concern. Further studies are warranted to elucidate *E. bieneusi* and *E. intestinalis* infection levels in meat and products derived from wild lagomorphs and the risk of transmission of these food-borne zoonotic pathogens.

## Figures and Tables

**Table 1:** Primers used for the detection of *Enterocytozoon bieneusi*, *Encephalitozoon intestinalis*, *Encephalitozoon hellem*, and *Encephalitozoon cuniculi*.

Microspodia Species	Primers	Forward Sequence (5′-3′)	Reverse Sequence (5′-3′)	Obtained Amplicon Size	Reference
*E. bieneusi*	EBIEF1/EBIER1	GAAACTTGTCCACTCCTTACG	CCATGCACCACTCCTGCCATT	607	[22]
*E. intestinalis*	SINTF/SINTR	TTTCGAGTGTAAAGGAGTCGA	TGCCATGCACTCACAGGCATC	822	[23]
*E. hellem*	EHELF/EHELR	TGAGAAGTAAGATGTTTAGCA	GTAAAAACACTCTCACACTCA	547	[24]
*E. cuniculi*	ECUNF/ECUNR	ATGAGAAGTGATGTGTGTGCG	TGCCATGCACTCACAGGCATC	549	[25]

**Table 2:** Prevalence of zoonotic microsporidia (*Enterocytozoon bieneusi*, *Encephalitozoon intestinalis*, *Encephalitozoon hellem*, and *Encephalitozoon cuniculi)* in organ meats from wild lagomorph species in Andalusia, southern Spain.

Variable	Categories	Prevalence (No. Positives/Overall) ^†^				
		Microsporidia	*E. bieneusi*	*E. intestinalis*	*E. hellem*	*E. cuniculi*
Species	Iberian hare	3.8 (3/79)	0 (0/79)	3.8 (3/79)	0 (0/79)	0 (0/79)
	Wild rabbit	1.0 (4/383)	0.8 (3/383)	0.3 (1/383)	0 (0/383)	0 (0/383)
Province	Cadiz	0 (0/56)	0 (0/56)	0 (0/56)	0 (0/56)	0 (0/56)
	Cordoba	7.0 (3/43)	2.3 (1/43)	4.6 (2/43)	0 (0/43)	0 (0/43)
	Jaen	2.4 (2/82)	1.2 (1/82)	1.2 (1/82)	0 (0/82)	0 (0/82)
	Almeria	0 (0/52)	0 (0/52)	0 (0/52)	0 (0/52)	0 (0/52)
	Malaga	0 (0/67)	0 (0/67)	0 (0/67)	0 (0/67)	0 (0/67)
	Seville	3.7 (2/54)	1.8 (1/54)	1.8 (1/54)	0 (0/54)	0 (0/54)
	Granada	0 (0/49)	0 (0/49)	0 (0/49)	0 (0/49)	0 (0/49)
	Huelva	0 (0/59)	0 (0/59)	0 (0/59)	0 (0/59)	0 (0/59)
Year	2015	1.2 (4/349)	0.9 (3/349)	0.3 (1/349)	0 (0/349)	0 (0/349)
	2016	0 (0/1)	0 (0/1)	0 (0/1)	0 (0/1)	0 (0/1)
	2017	0 (0/33)	0 (0/33)	0 (0/33)	0 (0/33)	0 (0/33)
	2018	3.8 (3/79)	0 (0/79)	3.8 (3/79)	0 (0/79)	0 (0/79)
Age	Yearling	5.0 (1/20)	0 (0/20)	5.0 (1/20)	0 (0/20)	0 (0/20)
	Subadult	1.6 (1/62)	1.6 (1/62)	0 (0/62)	0 (0/62)	0 (0/62)
	Adult	1.1 (4/366)	0.6 (2/366)	0.6 (2/366)	0 (0/366)	0 (0/366)
Sex	Female	0.9 (2/235)	0.4 (1/235)	0.4 (1/235)	0 (0/235)	0 (0/235)
	Male	1.9 (4/212)	0.9 (2/212)	0.9 (2/212)	0 (0/212)	0 (0/212)

^†^ Prevalences are depicted as percentages. Missing values omitted.
